# Self-Reported Nonadherence Predicts Changes of Medication after Discharge from Hospital in People with Parkinson's Disease

**DOI:** 10.1155/2020/4315489

**Published:** 2020-07-04

**Authors:** Francis Feldmann, Hannah M. Zipprich, Otto W. Witte, Tino Prell

**Affiliations:** ^1^Department of Neurology, Jena University Hospital, Am Klinikum 1, Jena 07747, Germany; ^2^Centre for Healthy Ageing, Jena University Hospital, Am Klinikum 1, Jena 07747, Germany

## Abstract

**Background:**

Medication is often changed after hospital discharge in people with Parkinson's disease (PD).

**Objective:**

This observational study aimed to describe changes in PD medication after discharge and explore their association with self-reported adherence and clinical parameters.

**Methods:**

During hospitalisation sociodemographic characteristics, the Movement Disorder Society-sponsored revision of the Unified PD Rating Scale for motor function (MDS-UPDRS III), Hoehn and Yahr (H&Y) stage, levodopa equivalent daily dose (LEDD), Beck Depression Inventory II (BDI-II) score, Montreal Cognitive Assessment (MoCA) score, nonmotor symptoms questionnaire (NMSQ), and Stendal Adherence to Medication Score (SAMS) were collected in 125 people with PD. A semistructured interview was conducted 1 month after discharge to determine the extent and reasons for medication changes.

**Results:**

Thirty-eight patients (30.4%) changed their PD medication after discharge. Most changes (20.8%) were performed by physicians while 9.6% of patients changed their medication by themselves due to side effects, missing effect of the medication, missing knowledge about the indication, running out of medication, or nonspecific reason. This led to decreased doses while changes by physicians resulted in both increase and decrease of doses as well as new drug prescription. Patients without changes, patients with changes performed by them, and patients with changes performed by physicians did not differ in age, disease duration, MDS-UPDRS III, LEDD, NMSQ, MoCA, BDI-II, gender, marital status, or education. However, patients who themselves made the changes were more likely to be nonadherent according to baseline SAMS. Patients who made changes after discharge had higher SAMS modification and forgetting subscores than patients without changes or with changes made by physicians.

**Conclusion:**

Both intended and unintended nonadherence occur in patients who change medication after discharge. The use of an adherence questionnaire during inpatient treatment may help detect patients with higher risk of changing medication after discharge.

## 1. Introduction

Parkinson's disease (PD) is the second most common neurodegenerative disorder. In the course of the disease, a plethora of motor and nonmotor symptoms require an individual, often complex medical treatment. During the course of the disease, many patients need modification of their medication while in the hospital [1]. During the period after hospital discharge, changes in prescribed medication often occur, such as changing the dose, stopping a medication, or initiating a new one [2, 3]. Changes in medication after discharge can be initiated by physicians in the outpatient setting as well as by patients. The changes by patients may be unintended (e.g., forgetting) or intended. For example, in intended changes, patients may decide not to refill medications or to change the dosage without consultation with their physicians, thus becoming nonadherent.

The majority of studies investigating medication changes after discharge have focussed on general or psychiatric hospital settings [4]. Little is known about medication changes in patients with PD after discharge from a movement disorder unit. Knowledge of this issue would improve our understanding of adherence to medication for PD, which is highly relevant because nonadherence to medication is associated with poor outcome, worsening of physical function, poor quality of life, and higher hospital admission rates [5–8].

For this purpose, self-reported adherence to medication questionnaire, as well as several clinical parameters, were assessed in patients with PD during their stay in the hospital. A short telephone interview was performed 1 month after discharge from hospital to determine the extent and reasons for changes in medication. We aimed to answer the following questions: how often was PD medication changed after discharge? Who changed the PD medication? How are changes made by patients related to clinical and sociodemographic parameters, such as adherence, depression, and educational level?

## 2. Materials and Methods

### 2.1. Design and Assessments

This study was approved by the local ethics committee of the Jena University Hospital (approval number 4572-10/15). All subjects gave written informed consent in accordance with the Declaration of Helsinki. The inclusion criterion was a diagnosis of PD according to the Movement Disorder Society (MDS) diagnostic criteria. Exclusion criteria were as follows: inability to fulfil a questionnaire, delirium. The reasons for admission were, among others, increase in fluctuations, worsening of dyskinesias, increase in off-phases, and worsening of gait and freezing. All tests were conducted during the medication on-phase. The data were collected between October 2018 and March 2019.

The demographic data collected included age, gender, marital status (single, divorced/widowed, or married), level of education (high: German *Abitur* or university; medium: German *Realschule* or the General Certificate of Secondary Education; low: German *Hauptschule* or no school), and employment status. Several clinical parameters were recorded: current prescribed medication, levodopa equivalent daily dose (LEDD) [9], the MDS-sponsored revision of the Unified PD Rating Scale III (MDS-UPDRS III; motor function), the revised nonmotor symptoms questionnaire (NMSQ), and Hoehn and Yahr (H&Y) staging. Cognition was assessed by the Montreal Cognitive Assessment (MoCA) score [10]. Beck Depression Inventory II (BDI-II) was used to quantify depressive mood. Adherence was assessed using the German Stendal Adherence to Medication Score (SAMS). The SAMS is based on 18 questions forming a cumulative scale (0–72), in which 0 indicates complete adherence and 72 indicates complete nonadherence [11]. The SAMS allows the assessment of three common reasons for/clusters of nonadherence: modification of medications, forgetting to take the medications, and missing knowledge about the medications [12]. The entire copy of the SAMS is available online (CC BY NC 3.0 licence; https://data.mendeley.com/datasets/ny2krr3vgg/1) [13].

The follow-up was conducted by a semistructured telephone interview 4 weeks after discharge, which included the following questions:

1. Was the prescribed PD medication changed since discharge from hospital? (yes; no)
2. What was changed? (decrease in dosage, increase in dosage, change of drug, and change in medication not specified)
3. Who changed the medication? (general practitioner, outpatient neurologist, and patient)
4. Why was the medication changed? (medication ran out or no prescription, missing or insufficient effect, missing knowledge about the indication, side effects, and others)

Up to three attempts were made to call the patients by telephone. The total number of patients recruited for the study was 130. A follow-up interview could not be performed for five patients. Therefore, 125 patients were included in the following analyses. For 12 patients who were not able to provide correct information, the caregivers were interviewed. These 12 patients did not differ from the remaining 113 participants in terms of age (*p*=0.15), LEDD (*p*=0.22), MoCa (*p*=0.24), MDS-UPDRS III (*p*=0.37), BDI (*p*=0.59), disease duration (*p*=0.43), and SAMS (*p*=0.44).

### 2.2. Statistical Analysis

SPSS (version 23.0, IBM Corporation, Armonk, NY, USA) was used for statistical analyses. Descriptive analyses were used to describe the cohort. The cutoff for nonadherence was set at a total SAMS >11 points (80th percentile) because it is generally considered that nonadherence becomes clinically significant when <80% of a prescribed medication is taken [14, 15]. The cohort was categorised into adherent (SAMS = 0), moderately nonadherent (SAMS = 1–11), and nonadherent (SAMS >11) patients. In addition, patients were categorised into three groups: without change in medication, with change initiated by the patient, and with change initiated by the physician. Comparisons of clinical and sociodemographic parameters between the groups were performed by analysis of variance, the Kruskal–Wallis test, and the chi-square test. Binary logistic regression was performed with change in medication (yes/no) as the dependent variable and SAMS and MDS-UPDRS III as the independent variables.

Based on our previous work, each patient was categorised into one cluster of nonadherence: modifications, missing knowledge, and forgetfulness [12]. For this purpose, the items that belonged to a cluster were summed up and divided by the number of items. This resulted in three mean values per SAMS cluster (called SAMS subscores). In the cluster “modifications,” medications were changed by the patients without consulting their doctors about either experiencing side effects or improvement in health. The cluster “missing knowledge” included patients who were unaware of the purpose and/or the dosage of their medication. The cluster “forgetfulness” included patients who frequently forgot to take their medication.

Normally distributed values are expressed as mean and standard deviation (SD); skewed values are expressed as median and interquartile range (IQR). All categorical variables are presented as numbers and percentages.

## 3. Results

### 3.1. Adherence at Baseline Investigation

Detailed characteristics of the 125 subjects are given in Table 1. According to the SAMS, 20 patients (16%) were categorised as fully adherent, 79 (63.2%) as moderately nonadherent, and 26 (20.8%) as nonadherent. The mean total SAMS was 6.4 (SD: 6.7; range: 0–41).

Thirty-seven participants reported at baseline that other people (caregiver and pharmacist) help with managing the medication (i.e., putting the pills into the pill box). The people in whom medication was self‐administered and people who need help did not differ in terms of age (*p*=0.10), LEDD (*p*=0.14), MoCa (*p*=0.42), disease duration (*p*=0.23), and SAMS (*p*=0.57). However, patients who need help for managing medication had a higher MDS-UPDRS III (*M* = 29.9; 95% CI: 25.9–33.9; *p* < 0.001) and more depression (BDI *M* = 15.3; 95% CI: 11.5–19.1; *p*=0.04) than patients who handle their medication independently (MDS-UPDRS III *M* = 21.5; 95% CI: 19.4–23.6; BDI *M* = 10.9, 95% CI: 9.5–12.4).

**Figure 1:**
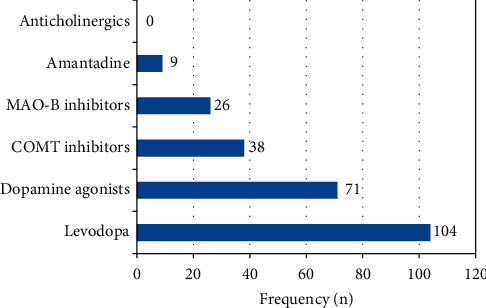
Number and classes of prescribed Parkinson's disease medication (*N* = 125).

Levodopa, dopamine agonists, and catechol-O-methyltransferase (COMT) inhibitors were the most frequently prescribed PD medication groups in our cohort (Figure 1).

### 3.2. Patterns and Reasons for Changes in Medication after Discharge from Hospital

**Figure 2:**
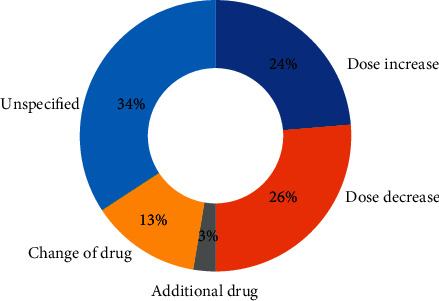
Types of medication changes in Parkinson's disease patients after discharge.

In 38 patients (30.4%), the PD medication was changed after discharge from hospital. The detailed changes are given in Figure 2. Medications were changed by patients (*n* = 12, 9.6% of the entire cohort) or physicians (*n* = 26, 20.8%), in particular by general practitioners (*n* = 9) and neurologists (*n* = 17). Of note, 11 of the 38 changes made by physicians were recommended at discharge in the discharge letter. Therefore, an unintended change in medication was observed in 27 patients (21.6%) only.

**Figure 3:**
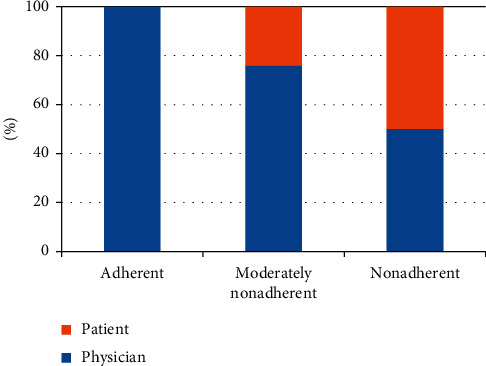
Initiators of medication changes in Parkinson's disease patients after discharge.

When the change was initiated by the patient, the dose was usually decreased (Table 2). In contrast, when the change was initiated by the physician, the dose could be either increased or decreased. New drugs were prescribed only by neurologists and not by general practitioners (Table 2). The reasons for changes in medication were side effects (*n* = 10), missing effect of the drug (*n* = 8), missing knowledge about the indication/necessaries (*n* = 3), and running out of the prescribed medication (*n* = 1); 8 patients were not able to name a specific reason for the change (they did not know why the change was made), and 8 patients reported other unspecific reasons for changing medication. The dose was significantly more likely to be increased when “missing effect” was the reason for changing the medication and was significantly more likely to be decreased when “side effects” were the reason for changing the medication (*p*=0.07, chi-square test) (Table 3). When patients were grouped according to their baseline adherence, we found that with a higher degree of nonadherence (higher SAMS), the change in medication was more frequently performed by the patient and not by the physician (*p*=0.01, chi-square test) (Figure 3).

### 3.3. Predictors of Changes in Medication after Discharge from Hospital

Patients with and without changes in PD medication were comparable in age (*p*=0.42), disease duration (*p*=0.75), LEDD (*p*=0.20), MoCA score (*p*=0.66), and BDI-II (*p*=0.58). Patients with and without changes in medication also did not differ in gender (*p*=0.45), marital status (*p*=0.71), educational level (*p*=0.29), or occupation (*p*=0.81). However, patients with changes in medication had a (nonsignificantly) higher MDS-UPDRS III (*M* = 27.8; SD = 12.4; 95% CI: 23.7–31.9), compared with patients without changes (*M* = 22.7; SD = 10.2; 95% CI: 20.5–24.9).

The cohort was then categorised into patients without changes, patients with changes made by them, and patients with changes made by physicians. These three groups did not differ in age, disease duration, LEDD, NMSQ, MoCA score, BDI-II, gender, marital status, education, or occupation (Table 4). However, patients who made the changes by themselves were characterised by a (nonsignificantly) higher MDS-UPDRS III and a significantly higher SAMS at baseline investigation. This indicates that patients who changed medication after discharge were more likely to be nonadherent. Correspondingly, the group of patients who made the changes by themselves contained more nonadherent patients than the group of patients without changes or with changes made by physicians (*p*=0.01, chi-square test). A logistic regression analysis was performed with changes made by the patient (yes/no) as the dependent variable and MDS-UPDRS III and SAMS as the independent variables. Patient-performed medication change was associated with SAMS (OR = –0.15; *p*=0.001), but not with MDS-UPDRS III (*p*=0.55) (Nagelkerke's *R*^2^ = 0.23; *p* < 0.001).

Patients who decreased the dose (*n* = 7; SAMS = 20.3; SD = 12.3) or had side effects (*n* = 4; SAMS = 17.3; SD = 16.0) were more likely to be nonadherent than patients who increased the dose (*n* = 2; SAMS = 6.5; SD = 4.9) or experienced an insufficient effect (*n* = 2; SAMS = 6.5; SD = 4.9). However, the sample size for this subgroup analysis was low. Therefore, no confirmatory tests were performed, and this finding has to be interpreted with caution.

Knowledge about the prescribed PD medication (according to the corresponding SAMS subscore) did not differ among the three groups. However, patients who changed medication by themselves after discharge scored higher in the SAMS modification subscore and SAMS forgetting subscore than patients in the other groups. This indicates that both intended and unintended nonadherence were present in patients who changed medication by themselves (Table 4).

## 4. Discussion

The aim of this study was to describe the association between adherence to PD medication and changes in medication in a cohort of patients with PD who were discharged from a neurological ward. After discharge from hospital, the prescribed medication of nearly 40% of the patients was changed. In 11 cases (8.8%), the change was intended, i.e., recommended in the discharge letter, and in 27 cases (21.6%), the change was not recommended in the discharge letter. Both higher [1, 16] and lower [17] rates of medication change after hospital discharge can be found in the literature. These different results can be partly explained by the duration of the follow-up period. Our study and Mansur's study [18] detected medication changes 1 month after discharge with a similar rate of 40%. Other studies identified changes within 24–72 hours [17] and 48 hours [16] after discharge with very different rates of 14.1% and 56%.

In our study, the most common reason for change in medication was side effects. Side effects were frequently associated with dose decrease performed by patients. In our cohort, adherence was worse in patients who reported side effects as the cause of medication changes than in patients who did not report side effects as the cause of medication changes. However, this association is not conclusive due to the small sample size in these subcohorts. In our study, we did not explore in detail the kind of side effects. This would be of interest in terms of drug safety and readmission to hospitals in future studies [19]. Missing or insufficient effect was the second most common reason for change in medication, and it was frequently associated with dose increase performed by physicians. In general, most changes in our study were performed by physicians. This confirms an earlier report that 70% of changes were made by physicians [18]. The high proportion of changes made by physicians also shows that patients with the disease severity like in our study (Table 1) perceive more regular visits to the neurologists than is the case with advanced PD [20].

In our study, changes in medication were not associated with disease severity, depression, cognitive function, or sociodemographic factors. Only self-reported nonadherence according to the SAMS was associated with changes in medication performed by patients. Changes made by physicians were not associated with poor adherence in the patients. The prevalence of nonadherence that we observed is in accordance with the results of previous epidemiological studies [18, 21, 22]. Patients who tend to modify or forget their medication (as indicated by higher corresponding SAMS subscores) are more likely to change medication after discharge. However, missing knowledge about prescribed medications (dosage, time, and reasons) was not associated with changes in medication after discharge. This is a remarkable finding and is in agreement with the study by Lindquist et al., in which senior patients with adequate health literacy, rather than those with inadequate health literacy, tended to purposely not adhere to postdischarge instructions [16].

What do these findings imply? In our opinion, the fact that in about 20% of cases, the medication was changed after inpatient treatment is relevant in several ways. First, it shows that side effects in patients with PD can occur with a certain delay and only become a problem when the patients are under their usual conditions at home. Adjustment of the dose by patients when they experience side effects is technically considered nonadherence, but it is actually a comprehensible and sensible reaction. A positive finding is that these patients only reduce the dose and do not immediately stop PD medication. Stopping the medication completely would certainly be associated with a more severe functional deterioration of motor function. For inpatient treatment, however, this also means that it does not make sense to increase dosages immediately before discharge because side effects would then occur with a delay at home. Unfortunately, this is exactly what is demanded by health insurance companies and the medical service of the health insurance companies in Germany, namely, the change in medication until the day of discharge, so that the treatment in the PD multimodal complex programme [23] can be fully billed. The second aspect, the extent to which nonadherent patients are more likely to perceive side effects (or whether side effects due to inadequate medication are more frequent) would have to be examined in further confirmatory studies with larger numbers of cases. Without doubt, the data also underline the importance of communication in the transition from inpatient to outpatient treatment [24–26]. However, the data also show that changes in medication after discharge are not only due to communication failure. Changes in medication were mainly dependent on the basic degree of adherence or nonadherence of the patient (already at baseline). Therefore, it remains to be clarified in further studies whether a different doctor-patient communication would reduce medication changes, or rather whether nonadherence is a variable of individual personalities that can only be influenced to a limited extent and is therefore always associated with a certain degree of intended or unintended medication change after discharge.

A noteworthy secondary finding of our study is the relationship between independent drug handling and cognition. It is generally assumed that people with dementia may need help in managing tablets. Conversely, however, our data indicate that people who do not manage tablets themselves automatically have cognitive limitations. There can be many reasons why people pass on the handling of medication to others, for example, fine motor limitations (higher MDS-UPDRS III), depression (higher BDI), or personal attitude.

This study is not free of limitations. Evidence for medication intake in PD patients is mostly based on self-reports [27]. Self-reported adherence questionnaires have been found to be insensitive for detecting suboptimal intake of PD medication [28]. However, only self-reports and interviews can reveal personal reasons for nonadherence. Methodologically, it should also be reflected that cognition and depression were not directly associated with drug changes. The fact that intentional and nonintentional nonadherence are influenced to different degrees by cognition and depression plays a role here. On the other hand, it can be assumed that the influence of cognition and depression is expressed differently when using a different method for measuring adherence (e.g., electronic pill count). This study included PD patients and PD medication only, and this should be kept in mind when extending the results to other groups of patients taking medication. Of course not all factors which contribute to medication changes could be assessed; e.g., comorbidities which could interfere with changes in PD medication should be considered in future trials. Although the cohort was comparable to that in other studies investigating adherence in PD patients [21, 29–33], the generalisability of the study is further limited by the selection process (only inpatients were included) and the low variation in sociodemographic characteristics of this group (mostly married, with middle-to-high education, and pensioned). Moreover, we only included patients who were able to understand and fulfil questionnaires. Therefore, our result cannot be generalised to people with severe cognitive impairment.

## 5. Conclusions

Self-reported nonadherence according to SAMS predicted changes in PD medication 1 month after hospital discharge. It remains to be seen whether intervention studies to improve adherence can reduce unintended changes in medication after discharge. The use of an adherence questionnaire during inpatient treatment may help to detect patients who are at higher risk of changing medication after discharge.

## Figures and Tables

**Table 1:** Clinical and demographic characteristics of the patients (*n* = 125).

Characteristic	Value	
	*N*	%
Gender		
Female	49	39.2
Male	76	60.8

Marital status		
Single	5	4.0
Married	99	79.2
Divorced/widowed	19	15.2
No data	2	1.6

Education		
Low	29	22.8
Middle	49	38.6
High	45	35.4
No data	4	3.1

Occupation		
Unemployed	2	1.6
Pensioned	114	91.2
Employed	9	7.2

	Mean	SD
Age (yr)	70.0	8.0
H&Y stage (median, IQR)	3.0	1.0
MDS-UPDRS III	24.3	11.2
LEDD (mg)	602.2	346.7
Disease duration (yr)	8.1	5.2
NMSQ	10.6	4.9
MoCA	23.5	2.9
BDI-II	12.5	8.8
SAMS total	6.4	6.7

H&Y, Hoehn and Yahr stage; MDS-UPDRS III, Movement Disorder Society-sponsored revision of the Unified Parkinson's Disease Rating Scale for motor function; LEDD, levodopa equivalent daily dose; NMSQ, nonmotor symptoms questionnaire; MoCA, Montreal Cognitive Assessment sum score; BDI-II, Beck Depression Inventory II; SAMS, Stendal Adherence to Medication Score.

**Table 2:** Type of medication change and the initiator of change (the number of patients).

Type of change	Who changed the medication after patient's discharge from hospital ?	
	Patient	Physician^*∗*^
Dose increase	2	7 (N 5, GP 2)
Dose decrease	7	8 (N 4, GP 4)
Change of medication	1	4 (N 4, GP 0)
Unspecified	2	7 (N 4, GP 3)

^*∗*^The first number indicates changes by neurologists (N) and the second number indicates changes by general practitioners (GP).

**Table 3:** Type of medication change and the reason for change (the number of patients).

Type of change	Reason for change					
	Medicine ran out	Missing effect	Missing knowledge	Side effects	Unknown	Others
Dose increase	0	6	0	1	1	1
Dose decrease	1	0	1	7	4	2
Change of medication	0	1	2	1	0	1
Unspecified	0	1	0	1	3	4

**Table 4:** Characteristics of patients with and without changes in medication.

Characteristic	No change in medication		Change by physician		Change by patient		*p*
	*N*	%	*N*	%	*N*	%	
Gender							
Female	36_a_	41.4	7_a_	26.9	6_a_	50.0	0.30
Male	51_a_	58.6	19_a_	73.1	6_a_	50.0	

Marital status							
Married	70_a_	81.4	20_a_	80.0	9_a_	75.0	0.83
Divorced/widowed	12_a_	14.0	4_a_	16.0	3_a_	25.0	
Single	4_a_	4.7	1_a_	4.0	0	0.0	

Educational level							
Low	18_a_	20.9	7_a_	28.0	4_a_	33.3	0.13
Middle	38_a_	44.2	5_a_	20.0	6_a_	50.0	
High	30_a_	34.9	13_a_	52.0	2_a_	16.7	

Employment status							
Pensioned	80_a_	92.0	23_a_	88.5	11_a_	91.7	0.88
Employed	6_a_	6.9	2_a_	7.7	1_a_	8.3	
Unemployed	1_a_	1.1	1_a_	3.8	0^2^	0.0	

	Mean	SD	Mean	SD	Mean	SD	
Age (yr)	70.4_a_	7.2	68.9_a_	10.4	69.5_a_	7.4	0.71
H&Y stage (median, IQR)	2.5 _a_	1	3.0 _a_	0.5	2.5 _a_	1.0	
MDS-UPDRS III	22.7_a_	10.2	28.0_a_	13.6	27.3_a_	10.2	0.06
LEDD (mg)	575.0_a_	343.4	620.9_a_	302.1	763.3_a_	446.2	0.23
Disease duration (yr)	8.2_a_	5.2	7.9_a_	5.8	7.9_a_	4.1	0.94
NMSQ	10.6_a_	4.6	11.2_a_	5.8	9.3_a_	4.8	0.54
MoCA	23.5_a_	2.9	23.6_a_	3.0	22.5_a_	3.4	0.56
BDI-II	12.2_a_	8.7	12.7_a_	9.5	14.2_a_	8.4	0.77
SAMS total	5.1_a_	5.3	6.8_a_	5.1	14.5_b_	11.9	<0.001
SAMS subscore missing knowledge	0.4_a_	0.8	0.5_a_	0.7	0.7_a_	0.8	0.36
SAMS subscore modification	0.1_a_	0.2	0.1_a_	0.2	0.7_b_	1.2	<0.001
SAMS subscore forgetting	0.4_a_	0.5	0.5_a_	0.4	1.3_b_	1.1	<0.001

Values in the same row and subtable where the subscripts are not identical differ greatly at *p* < 0.05 in the two-sided test for equality for column portions. With the help of the Bonferroni correction, the tests are adapted to all pairwise comparisons within a line of the innermost subtable.

## Data Availability

The data of this study are available from Mendeley Data: Prell, Tino (2020), “Changes of medication after discharge from hospital in people with Parkinson's Disease,” Mendeley Data, V1 (doi: 10.17632/jfxsrzv98y.1) (https://data.mendeley.com/datasets/jfxsrzv98y/draft?a=7d6b3606-326b-46ff-9b80-ab235534b468).
